# Sliding Crosslinked Thermoresponsive Materials: Polypseudorotaxanes Made of Poly(N-Isopropylacrylamide) and Acrylamide-γ-Cyclodextrin

**DOI:** 10.3389/fchem.2018.00585

**Published:** 2018-11-23

**Authors:** Giulio Malucelli, Jvan Dore, Davide Sanna, Daniele Nuvoli, Mariella Rassu, Alberto Mariani, Valeria Alzari

**Affiliations:** ^1^Department of Applied Science and Technology, Institute of Materials Science and Engineering for Innovative Technologies, Politecnico di Torino, Alessandria, Italy; ^2^Department of Chemistry and Pharmacy, University of Sassari, Sassari, Italy

**Keywords:** thermoresponsive materials, polypseudorotaxane, PNIPAAm, cyclodextrin, hydrogel, LCST

## Abstract

Novel polypseudorotaxanes (PPR) based on poly(N-isopropylacrylamide) (PNIPAAm) and acrylamide-γ-cyclodextrin (AγCD) are successfully synthesized. AγCD gives rise to sliding crosslinking systems and influences the thermoresponsive and swelling behavior of PNIPAAm hydrogels. Namely, their lower critical solution temperature (LCST) can be tuned up to 38°C, thus making the resulting materials of great interest in biomedical applications. Also, AγCD influences the thermal and mechanical properties of hydrogels, by affecting the *T*_g_ and *E* modulus values.

## Introduction

Polyrotaxanes (PRs) are among the most thoroughly investigated types of supramolecular polymers, consisting of one or more cyclic molecules threaded onto a linear polymer axis and end-capped with bulky moieties on both terminals of the macromolecular chain (Harada et al., 1999, 2009; Huang and Gibson, 2005; Wenz et al., 2006; Araki and Ito, 2007; Li and Loh, 2008).

Cyclodextrins (CDs) are ring-shaped molecules obtained by degradation of starch, composed of five or more 1-4-linked α-D-glucopyranoside units, as in amylose. Thanks to their non-toxicity, not absorbability in the upper gastrointestinal tract and their capacity to be metabolized by the colon microflora, their main use is in food industry (Del Valle, 2004; Marques, 2010). The CD structure is made of hydrophilic and hydrophobic domains: the former are located in the exterior part and bear polar groups that allow solvating CD molecules in aqueous media; the latter are located in the interior, non-polar part, and enable encapsulation of molecules or moieties having analogous polarity: this feature has been thoroughly explored in supramolecular, pharmaceutical, and analytical chemistry (Hedges, 1998; Nepogodiev and Stoddart, 1998; Ammar et al., 2012; Machín et al., 2012; Bazhban et al., 2013; Kaceriakova and Spanik, 2014; Khodaverdi et al., 2014; Silva et al., 2014; Hartlieb et al., 2017). In their ring, the most commonly used CDs contain a number of glucose units ranging from 6 to 8, forming a cone shape. Namely, α-CDs, β-CDs, and γ-CDs are 6-, 7-, and 8-membered sugar ring molecules, respectively.

Since the first discovery of PRs, synthesized from α-CDs with poly(ethylene glycol) (PEG) by Harada and Kamachi (1990), several studies have been carried out to design and prepare various advanced architectural CD-based PRs (Taylor et al., 2000; Sarvothaman and Ritter, 2004; Tong et al., 2009): in fact, CDs can be selectively self-assembled with a number of polymers and readily functionalized by a variety of synthetic strategies (Miljanić et al., 2007; Fernando et al., 2016).

In the latest years, the research community has paid great attention to the encapsulation of monomers and polymers in CDs, in particular in the field of smart or stimuli responsive hydrogels. These latter are polymeric materials able to swell or deswell as a function of external stimuli (Liu and Fan, 2002; Kopecek, 2007; Neal and Goldup, 2014). One of the most studied stimuli-responsive polymer is poly(N-isopropylacrylamide) (PNIPAAm), which is a non-toxic, biocompatible, and relatively cheap polymer characterized by a lower critical solution temperature (LCST) at about 30–32°C. These properties make PNIPAAm suitable for biomedical and pharmaceutical applications. However, its poor thermal and mechanical properties greatly limit its application. Moreover, the possibility of increasing its LCST up to the physiological range could provide great practical advantages. PRs comprising γ-CDs as host molecules and PNIPAAM, as a guest polymer, were prepared via self-assembly in aqueous solution (Zhang et al., 2008; Wang et al., 2011). Li and Loh (2008); Zhang et al. (2008) designed and synthesized β-CD-(PNIPAAm)_4_ in the presence of adamantyl-containing PEGs. The thermosensitive behavior of the β-CD-core star PNIPAM in the block copolymers was affected significantly, depending on the ratio of the adamantyl moiety to the β-CD core and/or the length of the PEG blocks, providing an efficient way to control the LCST. Therefore, this supramolecular approach might be promising for the production of intelligent systems for biomedical and pharmaceutical applications.

Recently, our research group investigated the effects of CDs on the properties of poly(2-hydroxyethylacrylate) hydrogel obtained by frontal polymerization (FP) (Nuvoli et al., 2016) and synthesized β-CD-based supramolecular hydrogels by FP, using acryloyl-β-cyclodextrin (AβCD) as the NIPAAM comonomer (Sanna et al., 2017). It should be highlighted that, due to the bulky pendant isopropylamide group, PNIPAAM cannot freely enter and slide through α-CD or β-CD cavities (Zhang et al., 2010; Li et al., 2016). However, we reasoned that this might happen if γ-CD is used, thus giving rise to a polypseudorotaxane (PPR) (Wang et al., 2012).

In this context, in the present work, a novel PPR system based on PNIPAAm and acrylamide-γ-cyclodextrin (AγCD) has been synthesized: CD acts as a pendant group and, at the same time, it may allowPNIPAAm macromolecular chains to flow through the empty cavity of CDs. As a consequence, a sliding crosslinking system is created, which should be characterized by peculiar swelling behavior and mechanical features different from those characterizing the conventional, covalently crosslinked analogous materials.

The thermal properties of resulting PPR hydrogels have been assessed through differential scanning calorimetry (DSC). The LCST of the hydrogels has been evaluated as well, exploiting swelling measurements at different temperatures. Finally, the mechanical behavior of the PPR systems has been assessed through compression and cyclic compression tests: these latter were also exploited for supporting the hypothesis that CD acts as a sliding, non-covalent crosslinking agent.

## Materials and methods

### Materials

Ammonium persulfate (APS, MW = 228.20), N-isopropylacrylamide (NIPAAm, MW = 113.16), N,N′-methylene-bis-acrylamide (MBA, MW = 154.17), tetramethylenediamine (TEMED, MW = 116.20, *d* = 0.775 g/ml);triethylamine (MW = 101.19, *d* = 0.796 g/mL) acryloyl chloride (MW = 90.51, *d* = 1.114 g/mL) were purchased from Sigma Aldrich. N-methyl pyrrolidone (NMP) was purchased from Sigma-Aldrich and distilled on molecular sieves before use. γ-cyclodextrin (γ-CD, MW: 972.89) was kindly supplied by IMCD Italia S.p.A. Mono-6-amino-deoxy-6-γ-cyclodextrin was obtained according to the literature (MW: 971.89, Tang and Ng, 2008).

### Synthesis of acrylamide-γ-cyclodextrin

Fifteen grams of mono-6-amino-deoxy-6-γ-cyclodextrin (0.015 mol), previously dried in oven at 60°C for 1 h, were put in a 250 ml round bottom flask with 200 ml of dry N-methylpyrrolidone and dissolved through vigorous stirring at room temperature. Then, 1.9 ml of triethylamine (0.015 mol) were added. The mixture was cooled down to 0°C, and 1.22 ml (0.015 mol) of acryloyl chloride were added dropwise under stirring in argon atmosphere. The system was left overnight at room temperature. After, 400 ml of cold methanol were added to precipitate triethylammonium chloride, which was eventually eliminated by centrifugation. The resulting liquid phase was poured in a large amount of acetone, in order to precipitate the desired product. The obtained white solid, AγCD (MW = 1,024.89), was recovered by centrifugation and washed three times with acetone to eliminate residual NMP. Yield: 36% (white-pale yellow powder).

$$\begin{array}{l} -{\;}^{1}\text{HNMR }\left({\text{D}}_{2}\text{O }400\text{ MHz}\right),\delta :6.46-5.92\left({\text{C}}_{\alpha }\text{H}={\text{C}}_{\beta }{\text{H}}_{2}\right),\\ \;\delta :5.04\left({\text{C}}_{1}\text{H}\right)\\ -{\;}^{1}\text{HNMR }\left(\text{DMSO }400\text{ MHz}\right),\delta :6.37-5.21\left({\text{C}}_{\alpha }\text{H}={\text{C}}_{\beta }{\text{H}}_{2}\right),\\ \;\delta :5.79\left({\text{C}}_{1}\text{H}\right) \end{array}$$

### Synthesis of PNIPAAm hydrogels containing AγCD

Various NIPAAm aqueous solutions (10% w/v) were put in a glass test tube, together with a known amount of AγCD (ranging from 0.5 to 2.0 mol% of AγCD with respect to the molar amount of NIPAAm). Subsequently, APS and TEMED were added (1.00:1.22 mol/mol, respectively), varying the APS concentration from 0.25 to 1.0 mol% (with respect to the total molar amount of NIPAAm and AγCD). The polymerization reaction was carried out at 4°C for 3 h. Yields were quantitative.

### Synthesis of PNIPAAm hydrogels containing γ-CD or MBA

NIPAAm aqueous solution (10% w/v) was mixed with 1 mol% (with respect to the molar amount of NIPAAm) of γCD in a glass test tube. Subsequently, APS and TEMED were added (1.00:1.22 mol/mol; 1 mol% of APS with respect to the total molar amount of NIPAAm and γCD). The polymerization reaction was performed at 4°C for 3 h (hereinafter coded as sample DγCD). The same materials and method were exploited for synthesizing PNIPAAm hydrogels containing MBA as a crosslinker, which replaces γCD (hereinafter coded as sample D_MBA_). The obtained hydrogels were washed several times in distilled water. The compositions of all the synthesized samples are listed in Table 1. Yields were quantitative.

**Table 1:** Composition, *T*_g_, LCST, and modulus values of the samples synthesized in the present work.

**Sample**	**AγCD (mol%)**	**APS (mol%)**	**T_g_ (^°^C)**	**LCST (^°^C)**	**E (MPa)**
A_0.5_	0.5	0.25	138	34	16.8
A_1_	1.0	0.25	152	35	21.8
A_2_	2.0	0.25	149	37	25.5
B_0.5_	0.5	0.50	148	35	11.9
B_1_	1.0	0.50	148	35	18.5
B_2_	2.0	0.50	153	37	21.3
C_0.5_	0.5	1.00	145	35	17.0
C_1_	1.0	1.00	152	35	16.0
C_2_	2.0	1.00	152	38	17.0
	γ-CD (mol%)			
D_γ*CD*_	1.0	1.00	–	–	–
	MBA (mol%)			
D_MBA_	1.0	1.00	124	32	–

### Differential scanning calorimetry

DSC analyses were performed by using a DSC Q100 Waters TA Instruments calorimeter, equipped with TA Universal Analysis 2000 software. Two types of analyses were performed. The first one was carried out on hydrogels immediately after synthesis. To this aim, the obtained products were subjected to a heat scan, from 0 to 250°C, with a heating rate of 20°C/min in inert atmosphere (nitrogen flow: 50 ml/min), in order to evaluate the conversion (Scognamillo et al., 2010). The second analysis was carried out on samples after being washed in water and dried in a vacuum oven at 50°C for 3 days: the resulting hydrogels were subjected to a heat/cool/heat cycle scan, from −80 to 250°C, with a heating rate of 20°C/min and a cooling rate of 10°C/min in inert atmosphere (nitrogen flow: 50 ml/min), in order to evaluate the glass transition temperature (*T*_g_).

### Swelling measurements

After synthesis, all samples were washed and swollen in distilled water for 2 weeks, in order to remove solvents and unreacted reagents and to reach swelling equilibrium. Then, they were cut into small pieces having similar shape and size, and immersed in distilled water in a thermostatic bath, in order to assess their behavior; the temperature of water, in which they were immersed, was varied from 20 to 40°C at a rate of 1°C/day. Their swelling behavior as a function of temperature was measured. The swelling ratio (SR%) of hydrogels was calculated according to the following equation (1):

(1) $$\begin{array}{r} SR\%=\frac{M_{s}-M_{d}}{M_{d}}*100 \end{array}$$

where *M*_*s*_ and *M*_*d*_ are the sample masses in the swollen and in the dry state, respectively. The LCST of the hydrogels was determined by the flex of the swelling curve in a plot of SR% as a function of temperature. The *M*_*d*_-value for each sample was determined, at the end of the experiment, on the samples dried in a vacuum oven at 50°C for 3 days.

### Compression tests

Compression tests were performed on cylindrical specimens (diameter: 15 mm, height: 10 mm), according to ISO 527-1 standard, using a Zwick-Roll Z010 apparatus, equipped with a 5 kN load cell, at 23°C and 50% relative humidity, applying 0.05 N pre-load; the speed test was 2 mm/min. At least five tests were carried out for each material in order to have reproducible and significant data. Furthermore, cyclic compression tests were carried out, using the same apparatus: for these tests, the load was increased from 0 to 3 N at 0.2 N/min and then decreased using the same force decrement.

## Results and discussion

**Figure 1:**
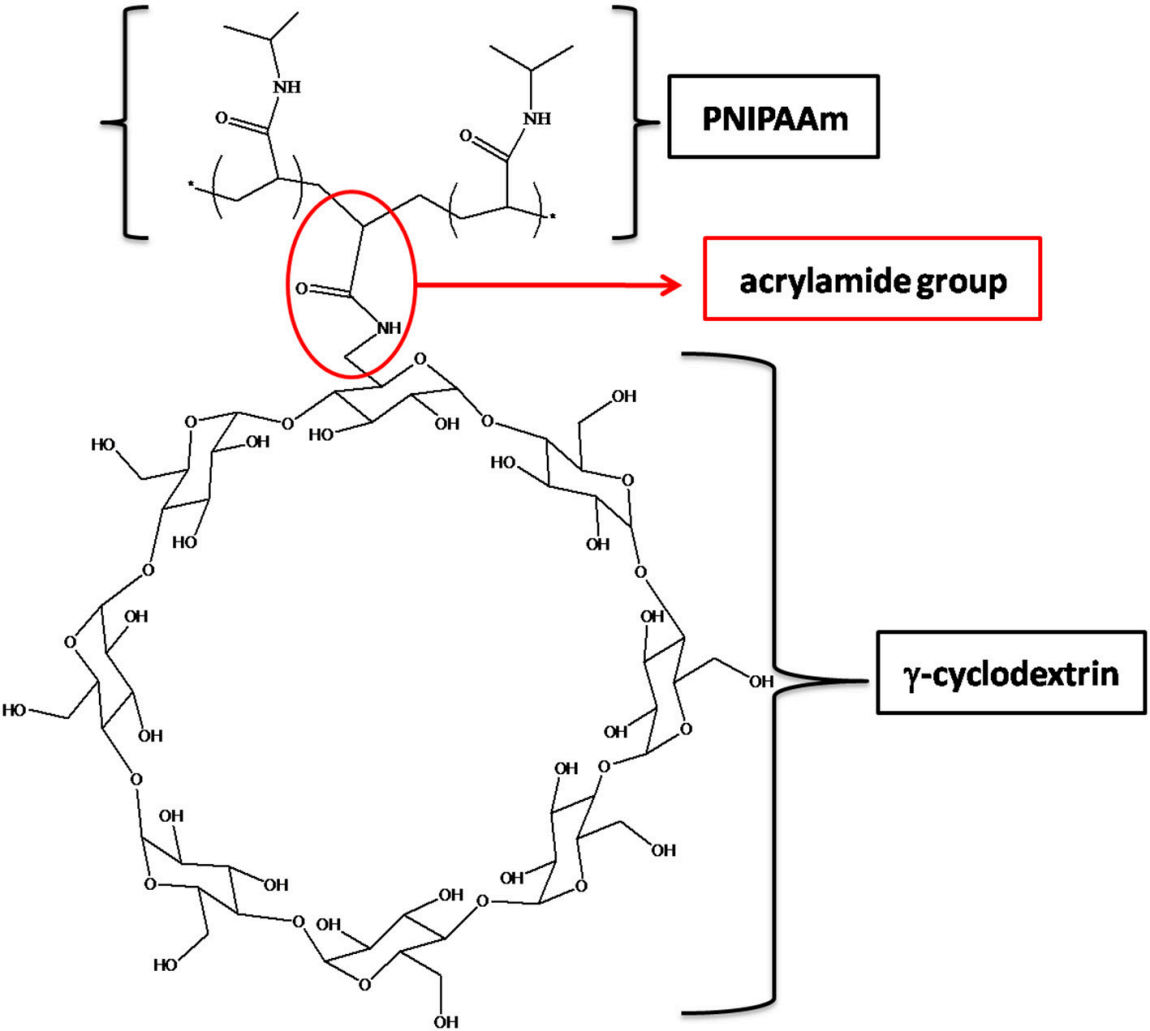
Structure of AγCD linked to a PNIPAAm macromolecular chain.

In order to prepare PNIPAM-based PPRs, γ-CD was used. It should be noticed that its α and β counterparts were not considered suitable for this purpose. This is due to the relatively large size of γ-CD cavity, which allows NIPAAm to enter, hence giving rise to the corresponding inclusion complex. In addition, after the polymerization reaction, γ-CD allows PNIPAAm macromolecular chains to easily slide through. Indeed, it is known that smaller cavity size CDs are not able to do that (Wang et al., 2012). This is very intriguing, since it may significantly affect the mechanical behavior of the resulting system. Besides, the γ-CD used in this work was monoacrylated, in order to act as a NIPAAm comonomer, being it eventually covalently linked to PNIPAAm chains. The resulting PPR system is constituted of macromolecular chains bearing CDs as pendant groups; at the same time, these chains are non-covalently crosslinked and characterized by non-covalent sliding crosslinking (Figure 1).

**Figure 2:**
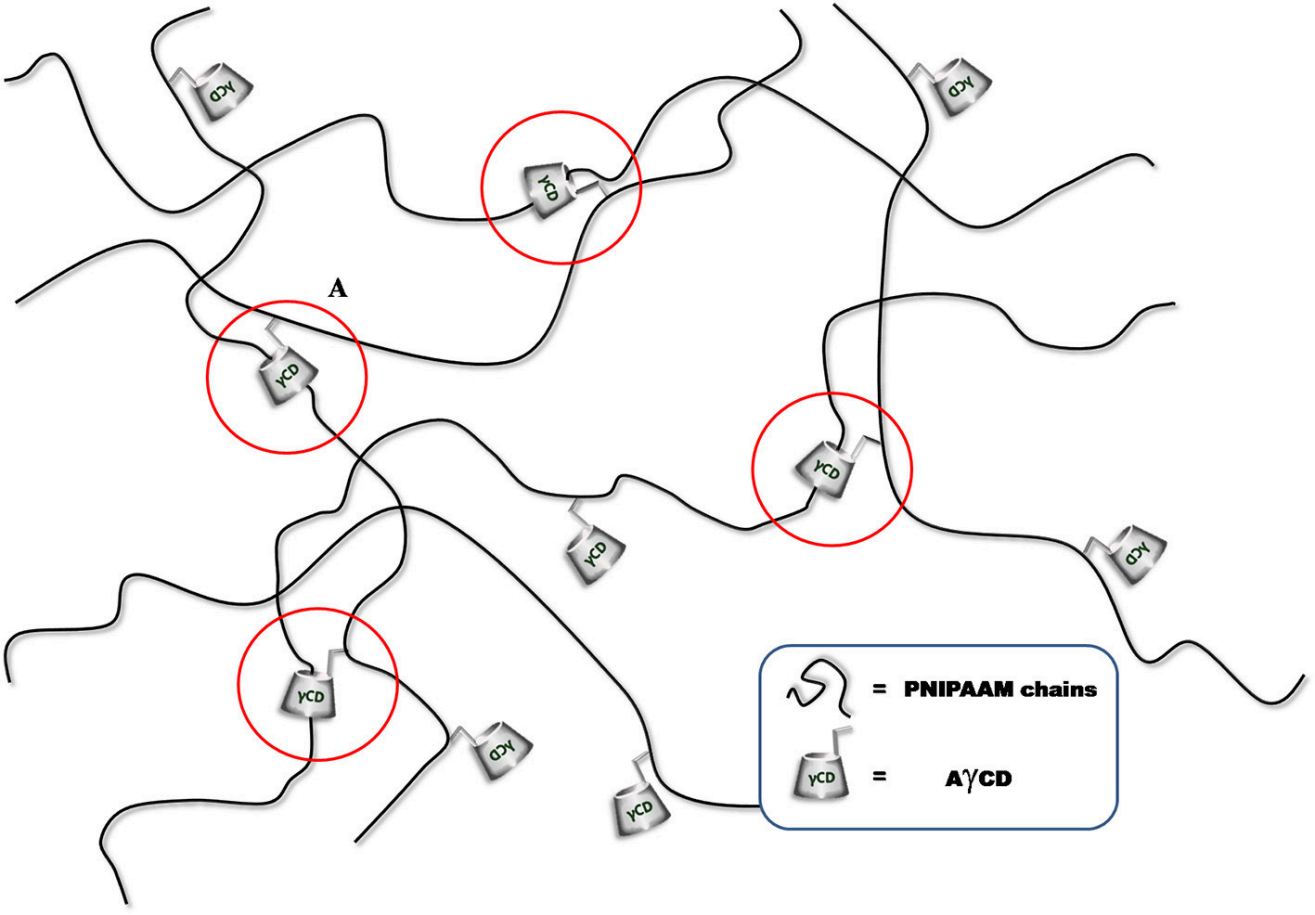
Schematic illustration of sliding crosslinking exerted by AγCD.

As a first evidence, we noticed that the resulting hydrogels swell without dissolving in water. Since AγCD is monoacrylated, it cannot act as a covalent crosslinker. However, the observed behavior of the hydrogels containing AγCD suggested us that a sort of crosslinking should actually be present, which may be attributed to the polypseudorotaxane formation (Figure 2).

On the basis of what reported in literature about elastomers, which typically contain ca. 1 mol% of crosslinker (Mooney, 1940; Flory and Rehner, 1943; Alemán et al., 2007) we chose similar amounts for AγCD in order to ensure a close degree of crosslinking, but resulting from non-covalent, sliding characteristics instead of covalent bonds. Thus, the concentration of AγCD was kept between 0.5 and 2 mol%, while that of initiator [I], was always between 0.25 and 1.0 mol%.

From a qualitative point of view, two opposite cases were considered:

1. when [I] and [AγCD] are, respectively, 0.25 and 2.0 mol%, the highest number of AγCD units linked to the macromolecular chains is present;
2. conversely, when [I] and [AγCD] are 1.00 and 0.5% mol, respectively, the lowest number of AγCD units is linked to the polymeric chains.

The reaction temperature was also a parameter that was taken into proper account. In particular, the polymerizations were carried out in water at 4°C in order to avoid solvent evaporation and bubble formation and to obtain hydrogels that are already in their swollen state (the LCST of PNIPAAm is at about 32°C) (Fujishige et al., 1989).

The composition and the *T*_g_, LCST, and modulus values (*E*) of the samples are listed in Table 1.

After synthesis, samples belonging to the A, B, and C series looked as colorless semi-solids, like the covalently cross-linked sample (D_MBA_). Once put in water, they just swell but without dissolving. On the contrary, the sample D_γ*CD*_, containing the unmodified γ-cyclodextrin only, looked physically different from the others: it was a colorless viscous liquid, completely miscible in water. This finding confirms that AγCD actually acts as a crosslinker.

### Thermal properties

DSC analyses performed on just-synthesized samples did not show any residual polymerization heat (exothermal), thus confirming that all monomer quantitatively converted into polymer.

In order to better characterize the thermal properties of hydrogels, DSC analyses were performed also on washed and subsequently dried samples. A heat/cool/heat cycle was performed on each sample (Table 1). A wide endothermic transition at about 160°C appeared during the first heating for all samples, which was attributed to the evaporation of water molecules from the cavity of AγCD. This transition was not recorded in the second heating ramp.

Furthermore, from the second heating, it was possible to evaluate the *T*_g_-values of polymers (Table 1). It was found that, as the AγCD amount increased, *T*_g_-values remarkably increased. Indeed, by comparing samples having the same amount of initiator, but a different amount of AγCD (ranging between 0.5 and 2 mol%), the *T*_g_-values raised from 138 to 153°C, respectively. In addition, by comparing the D_MBA_ sample with those containing AγCD, it was found that the first one exhibits significantly lower *T*_g_ (124°C). This finding can be easily explained by considering the decrease of free volume and the consequent reduction of macromolecular chain mobility exerted by CDs, which are rigid compounds able to exert strong polar interactions, through the formation of supramolecular aggregates (Valero et al., 2007; Jansook et al., 2010).

The amount of initiator did not influence the *T*_g_; in fact, by keeping constant the AγCD concentration and ranging the amount of APS from 0.25 to 1 mol%, the *T*_g_-values were almost unchanged.

Sample A_0.5_ showed a different behavior: in fact, its glass transition occurred at a temperature (i.e., 138°C) that is lower than that of all samples containing AγCD. This finding can be easily explained considering the qualitative approach mentioned above: sample A_0.5_ contains the lowest amounts of both AγCD and APS; thus, it is characterized by the longest macromolecular chains length and the lowest number of linked AγCD. As a consequence, its chain mobility is larger than that of the other samples. Anyhow, this value is higher than those of the “conventionally” crosslinked sample (D_MBA_, 124°C); this fact is probably due to the strong interactions between CD and hydrogel matrix.

### Swelling properties

**Figure 3:**
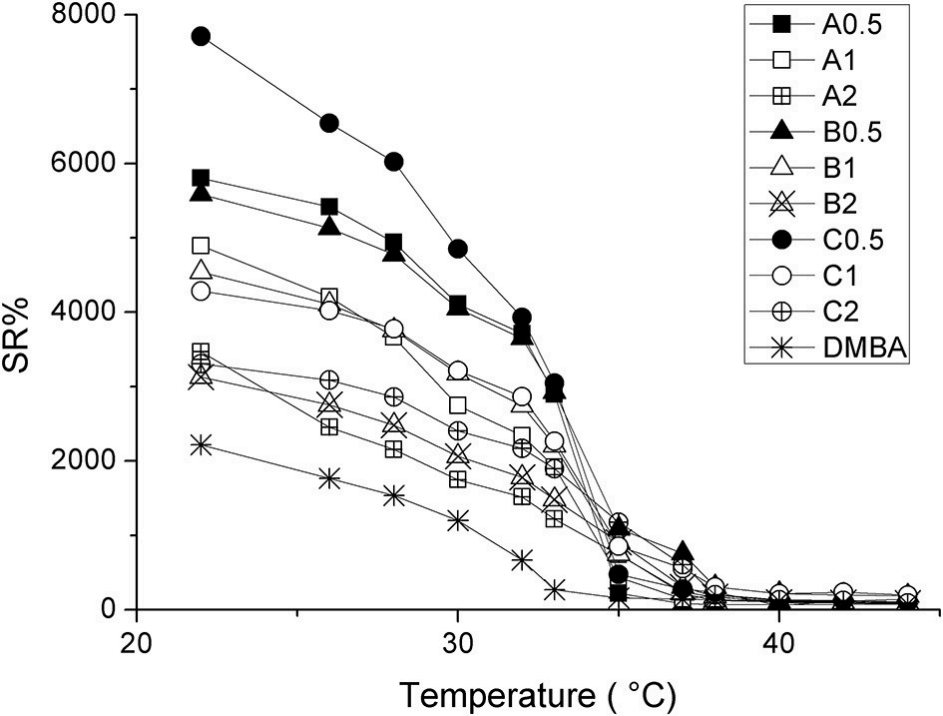
SR% as a function of temperature for synthesized samples, where squares, triangles and circles refer to A, B and C series, respectively. Amount of AγCD: 0.5 mol% (solid); 1.0 mol% (empty marker); 2.0 mol% (starred).

The influence of CDs on the swelling properties of PNIPAAm hydrogels was also evaluated: all the synthesized hydrogels were found to significantly swell in water; furthermore, the presence of AγCD influenced their SR% as a function of temperature (Figure 3).

In particular, as can be seen in Figure 3, the SR% increases by decreasing CD concentration, according to the following ranking: (A, B, C)_0.5_ > (A, B, C)_1_ > (A, B, C)_2_.

In detail, at 22°C, SR% goes from 7,800% for sample C_0.5_, to 3,100% for B_2_, hence demonstrating the large influence of AγCD on the swelling properties of PNIPAAm hydrogels. In addition, SR% value of D_MBA_ at 22°C is ca. 2,200%, a significantly lower value than that of CDs-containing samples. This is in agreement with the covalent nature of the crosslinker, which does not allow the chains to freely slide and swell (Karino et al., 2004; Zhao et al., 2005; Kopeček and Yang, 2007).

Furthermore, this is also a confirmation that the other samples are characterized by sliding crosslinking. By contrast, the effect of the amount of initiator was almost negligible.

AγCD influences also the thermoresponsive behavior of hydrogels. In fact, the “conventionally” crosslinked PNIPAAm (D_MBA_) shows the usual LCST at about 32°C (Fujishige et al., 1989), while hydrogels containing AγCD exhibit a higher value of LCST (Table 1). LCST-values increase from 35°C, for all the samples containing 0.5 mol% of CD (regardless of the amount of APS), to 38°C for those samples containing 2 mol% of AγCD. We consider this result of large interest for the possible practical applications of PNIPAAm also in the biomedical field, as the range of LCST variation can be tuned by including also the physiological body temperature. Moreover, this goal was reached by using non-toxic, largely available and cheap additives as CDs are.

### Mechanical properties

The mechanical properties of the slide-ring gel are quite different from those of conventional physical and chemical gels. Indeed, as reported in literature (Ito, 2007), the physical ones show a J-shaped stress-strain curve with large hysteresis, while the chemical gels show an S-shaped stress-strain curve (Ito, 2007). The large hysteresis in the physical gel is caused by recombination among non-covalent crosslinks in a polymer network on deformation.

**Figure 4:**
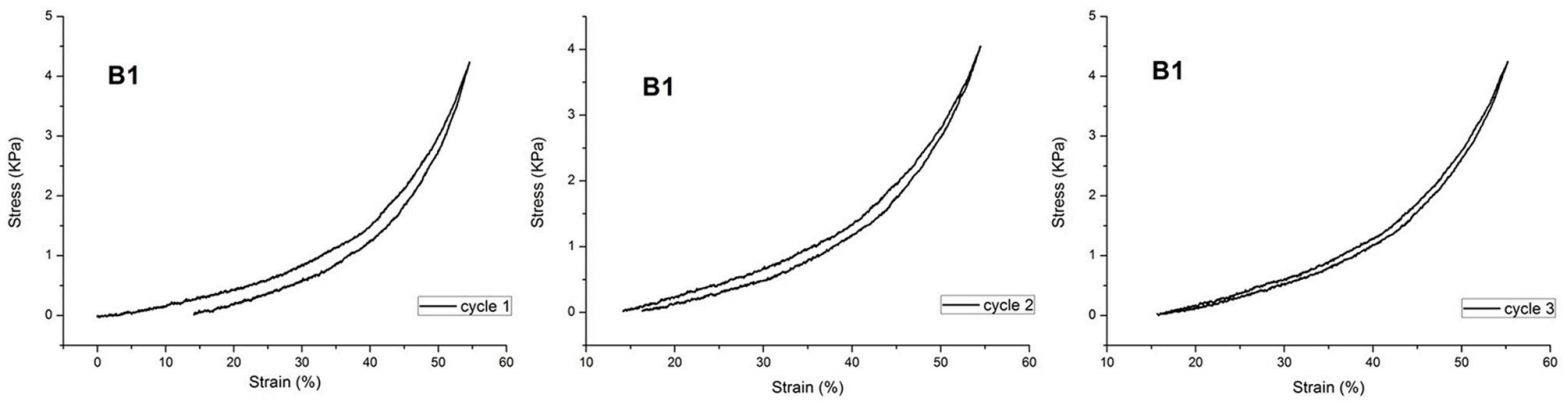
Cyclic compression curves for B_1_.

Actually, the polymeric hydrogels studied in this work exhibited a force/deformation curve characterized by a “J” shape without hysteresis (or very limited hysteresis phenomena; Figure 4), for B_1_, as an example.

The plotted curves refer to three sequential compression cycles carried out on each sample investigated. The slight shift shown after subsequent cycles can be due to the small water loss occurring during each compression. However, as far as the hysteresis is considered, the obtained values are very limited, also considering the water loss. This finding supports the hypothesis that CD acts as a sliding, non-covalent crosslinking agent (Ito, 2007). It was not possible to perform this characterization on sample D_MBA_ because of its high brittleness. Then, PNIPAAm/AγCD hydrogels were subjected to compression tests: as presented in Table 1, the compression modulus E increases with increasing the CD content. Even if these tests were performed on swollen (wet) samples, the results confirm what stated about the observed *T*_g_-increase in the dried sample. Indeed, both results can be attributable to the increase in crosslinking density. Furthermore, the presence of higher APS amounts in the hydrogel formulation turns out to lower the stiffness of the samples. This result confirms that, as the amount of initiator increases, the macromolecular chains are shorter: as a consequence, the probability that AγCD units are linked to the polymeric chains decreases. For the same reason reported above, the *E* modulus value of sample D_MBA_ could not be recorded.

## Conclusion

In this work, novel PPRs based on PNIPAAm and AγCD were successfully synthesized. AγCD acted as a pendant group and, at the same time, allowed macromolecular PNIPAAm chains to flow through its empty cavity, giving rise to a sliding crosslinking system with peculiar features. The amount of AγCD in the hydrogels was varied from 0.5 to 2 mol% in order to study the influence of CDs on hydrogel properties, such as swelling behavior, LCTS values and mechanical properties. In addition, the amount of initiator was varied from 0.25 to 1 mol%, in order to investigate its effect on the macromolecular chains length and on the probably that AγCD units can link to the polymeric chains. All the results were compared with covalent crosslinked samples (D_MBA_). All hydrogels were found to swell without dissolving in water; furthermore, since AγCD is monoacrylated and cannot act as a covalent crosslinker, this behavior suggests that a PPR structure is formed.

AγCD influenced the thermoresponsive behavior of PNIPAAm hydrogels, by modifying their LCST value that increased with increasing AγCD amount, from 32°C (D_MBA_ sample) to 38°C (C_2_ sample). This result, which approaches the LCST value of classical PNIPAAm to actual human body temperature, widens the practical applications of PNIPAAm in the biomedical field. It is also noteworthy that this goal was reached by using CDs, which are largely available, non-toxic, and cheap materials.

Furthermore, AγCD influenced also the swelling behavior of the resulting hydrogels; in fact, in particular, at temperatures below the LCST, the swelling of polymers increased with decreasing AγCD content. Besides, the presence of AγCD influenced the thermal and mechanical properties of hydrogels. In detail, the *T*_g_-values of samples increased with increasing AγCD content, owing to the decrease of free volume and the consequent reduction of macromolecular chain mobility exerted by the CDs. This result was in agreement with the observed increase of the compression modulus E of the hydrogels.

Finally, the synthesized hydrogels showed a force/deformation curve characterized by a “J” shape without or with a very limited hysteresis. This finding strongly supports the hypothesis that CD acts as a sliding, non-covalent crosslinking agent.

## Author contributions

GM: mechanical properties. JD: synthesis of modified cyclodextrin. DS: synthesis of mono-6-amino-deoxy-6-gamma-cyclodextrin. DN: swelling properties. MR: thermal properties. AM: coordinator of the work. VA: coordinator of the work and synthesis of hydrogel samples.

### Conflict of interest statement

The authors declare that the research was conducted in the absence of any commercial or financial relationships that could be construed as a potential conflict of interest.
